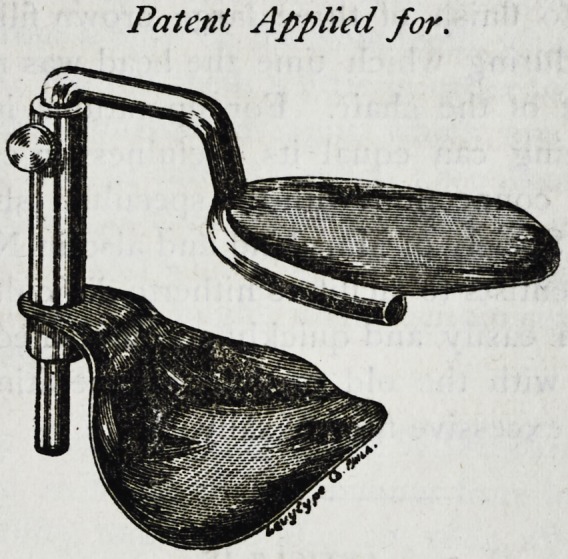# Mirror Topped Syphon Tongue Holder

**Published:** 1887-03

**Authors:** D. Genese

**Affiliations:** Baltimore, Md.


ARTICLE III.

### MIRROR TOPPED SYPHON TONGUE HOLDER.

INVENTED BY DR. D. GENESE, BALTIMORE, MD.

In preparing cavities in the lower jaw where a copious flow of viscid saliva is present, much time is lost and great inconvenience is felt by patient and operator. Absorbents fill the mouth and soon get useless and a pipe syphon irritates the tongue and keeps it continually rolling into the cavity one wants to prepare, while just as the patient is in a good position, the necessity occurs to empty the mouth and the operator looses precious time.

The rubber dam in such cases consumes much time, is often defective and moisture accumulates so fast under it that the patient gets distressed and often the clothes spoilt by the escape of saliva under the dam.

It is also a recognized fact that a plentiful supply of warm water to syringe cavities, getting rid of debris from the undercuts is the dentist's greatest safe-guard from troublesome marginal defects found so soon after completing fillings in mouths filled with viscid saliva, which almost glues the debris to the dentine. The posterior operations require good reflected light which cannot be obtained when the mouth is filled with cloths or rubber.

*Patent Applied for.*

This instrument enables such operations to be performed rapidly and with less fatigue to patient and operator. It keeps the tongue out of the way; shows a fine light upon the teeth and carries all fluid away as soon as formed on the floor of the mouth.

Under anæsthesia it will relieve the patient from the distress of swallowing the blood, the same in operations on cleft palate, or in spraying the fauces where the desire to swallow occurs almost as soon as the operation commences. It enables the dentist to have the cavity ready for plastic fillings before mixing the material and not to hurry the manipulation, thereby destroying its working properly by too much haste while the material is being introduced, and it *leaves both hands at liberty* without the fear of the tongue getting in the way.

It is made of german silver, nickle plated and highly finished, with three sizes of depressors, which will be found all that is required. It is easily kept clean and is rapidly handled. It can also be worked with a ball syringe, but a flow of water is preferred. It will syphon a pint per minute if desired, and by the arrangement of the inlets no undue pressure is felt on the soft tissues or the tongue during the operation. Its capacity has been tested in many cases, in one of which the patient retained it in the mouth from commencement to finish of three large crown fillings, taking two hours, during which time the head was not removed from the rest of the chair. For operations in children's mouths nothing can equal its usefulness in certain cases, and used in connection with the speculum shown at the State Dental Society of Maryland and also in New York, it will enable dentists to facilitate hitherto difficult operations, making them easily and quickly accomplished with better results than with the old method of operating in small mouths with excessive flow of saliva.

## Figures and Tables

**Figure f1:**